# A new species of *Harpacticella* Sars, 1908 (Copepoda, Harpacticoida), from a tidal pool on Jeju Island, Korea

**DOI:** 10.3897/zookeys.445.7831

**Published:** 2014-10-13

**Authors:** Seunghan Lee, Kichoon Kim, Wonchoel Lee

**Affiliations:** 1 Department of Life Science, Hanyang University, Seoul 133-791, Korea

**Keywords:** Harpacticidae, taxonomy, zoogeography, DNA barcode, marine, new species

## Abstract

A new species of the genus *Harpacticella* Sars, 1908 is described from a tidal pool on Jeju Island, Korea. *Harpacticella jejuensis* **sp. n.** is closely related to *Harpacticella itoi* Chang & Kim, 1991, with regard to the structure of P1 exp-1 and enp-1, the length of P1 exp-1 and exp-2, and the setal number of the P5 exopod in males. However, the new species is clearly distinguishable from *Harpacticella itoi* by the combined following characters: six setae on the P5 exopod in females, one naked seta on the inner margin of P1 exp-2, the short endopod of P1 compared to the exopod, and a naked long seta on the proximal inner margin of the P5 exopod of males. The mtCOI partial sequence is provided as a DNA barcode for the new species.

## Introduction

*Harpacticella* Sars, 1908 is a genus of harpacticoid copepods, family Harpacticidae Dana, 1846. The genus has been reported from various habitats (fresh water, brackish water, and marine), mostly in Asian waters (Itô and Kikuchi 1977; Chang and Kim 1991), and has also been recorded from the Pacific Northwest, USA (Cordell et al. 2007).

The first *Harpacticella* species report was by G.O. Sars (1908) who examined sandy littoral sediment of Lake Baikal. He proposed a new genus for this species, based on a reduced number of antennule segments and a two-segmented antennary exopod. So far, six species in the genus *Harpacticella* have been reported (Wells 2007). Among these species, *Harpacticella inopinata* Sars, 1908, *Harpacticella paradoxa* (Brehm, 1924), and *Harpacticella amurensis* Borutzky, 1952 were described from freshwater, *Harpacticella lacustris* Sewell, 1924 and *Harpacticella itoi* Chang & Kim, 1991 from brackish water, and *Harpacticella oceanica* Itô, 1977 from the marine environment.

During a study of the harpacticoid community along the coast of Jeju Island of Korea, we collected a new species of *Harpacticella* from a tidal pool. Here, we describe the new species and provide a key to species in the genus *Harpacticella*. Partial mtCOI sequence was also obtained as a DNA barcode for the new species.

## Materials and methods

Samples were collected by hand net (63 µm mesh-size) from a tidal pool on the coast of Jeju Island, Korea. Specimens were preserved in 99% ethanol. Specimens were dissected in lactic acid, and the dissected parts were mounted on slides with lactophenol mounting medium. Preparations were sealed with transparent nail varnish. All drawings were prepared using a drawing tube attached to an Olympus BX51 differential interference contrast microscope.

Descriptive terminology is adopted from Huys et al. (1996). Abbreviations used in the text are as follows: A1, antennule; A2, antenna; ae, aesthetasc; exp, exopod; enp, endopod; P1-P6, first to sixth thoracopod; exp (enp)-1(2,3), proximal (middle, distal) segment of a three-segment ramus; CR, caudal rami. Specimens were deposited in the National Institute of Biological Resources, Incheon, Korea (NIBR). Scale bars in figures are indicated in µm.

Molecular analysis. For DNA extraction, fixative materials (99% Et-OH) were removed from specimens by washing with distilled water, and DNA was extracted using a tissue DNA purification kit (COSMO GENETECH Co. Ltd., Korea). Amplifications were performed in 20 µl reactions volumes containing extracted tissue DNA, primers LCO-1490 (5’-GGT CAA CAA ATC ATA AAG ATA TTG G-3’) and HCO-2198 (5’-TAA ACT TCA GGG TGA CCA AAA AAT CA-3’) (Folmer et al. 1994), and PCR premix (BioNEER Co), using a TP600 thermal cycler (TAKARA). PCR conditions comprised initial denaturation at 94 °C for 300 s, followed by 40 cycles of denaturation at 94 °C for 60 s, annealing at 46 °C for 120 s, and extension at 72 °C for 180 s; a final extension step was then performed at 72 °C for 600 s. PCR products were evaluated by electrophoresing amplification products on a 1% agarose gel containing ethidium bromide. Purification of amplified products was performed using a PCR purification kit (COSMO GENETECH Co. Ltd., Korea), and both strands were sequenced using an ABI 3730XL sequencer (COSMO GENETECH Co. Ltd., Korea).

## Systematics

### Order Harpacticoida Sars, 1903 Family Harpacticidae Dana, 1846 Genus *Harpacticella* Sars, 1908

#### Harpacticella jejuensis sp. n.

Taxon classificationAnimaliaHarpacticoidaHarpacticidae

http://zoobank.org/77BE96F4-597C-44D0-A07E-03A4D993A5F2

**Figure 1.**
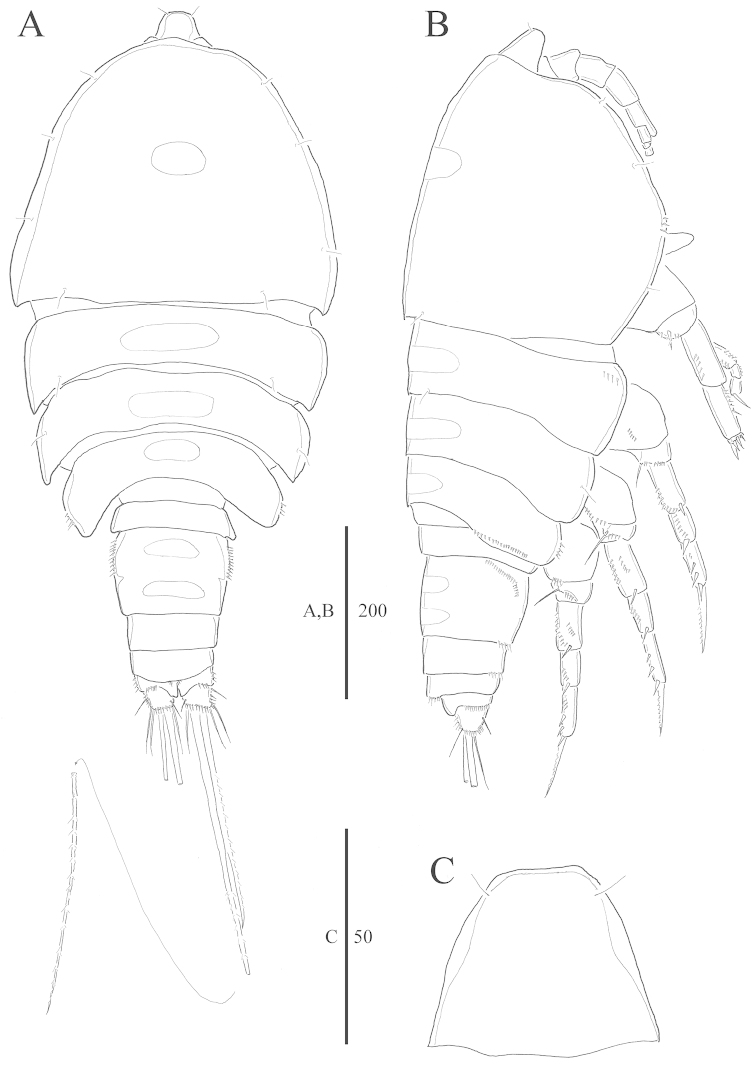
*Harpacticella jejuensis* sp. n. Female. **A** habitus, dorsal **B** habitus, lateral **C** rostrum. Scale bars in µm.

**Figure 2.**
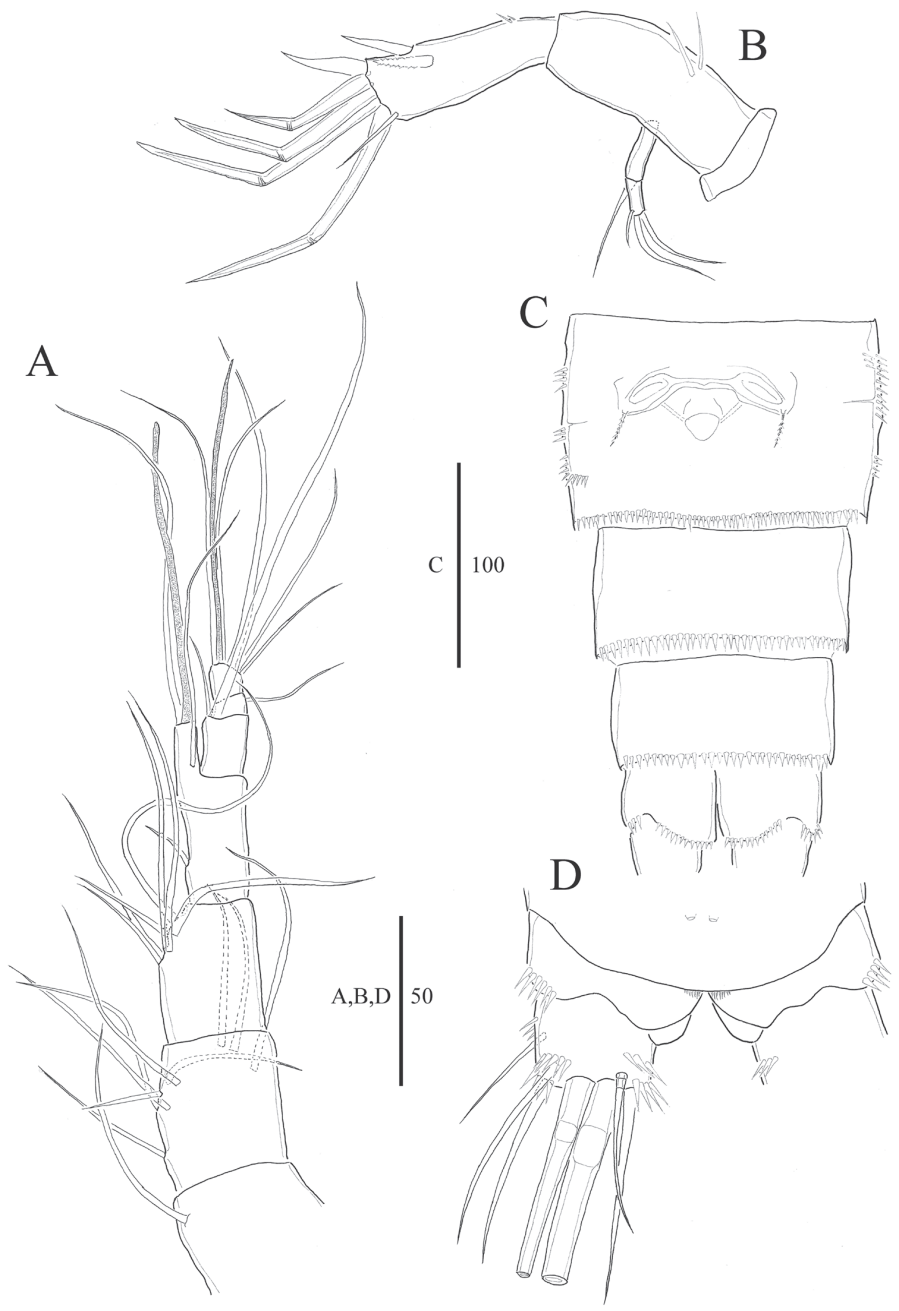
*Harpacticella jejuensis* sp. n. Female. **A** antennule **B** antenna **C** urosome (excluding P5-bearing somite), ventral **D** caudal rami, dorsal.

**Figure 3.**
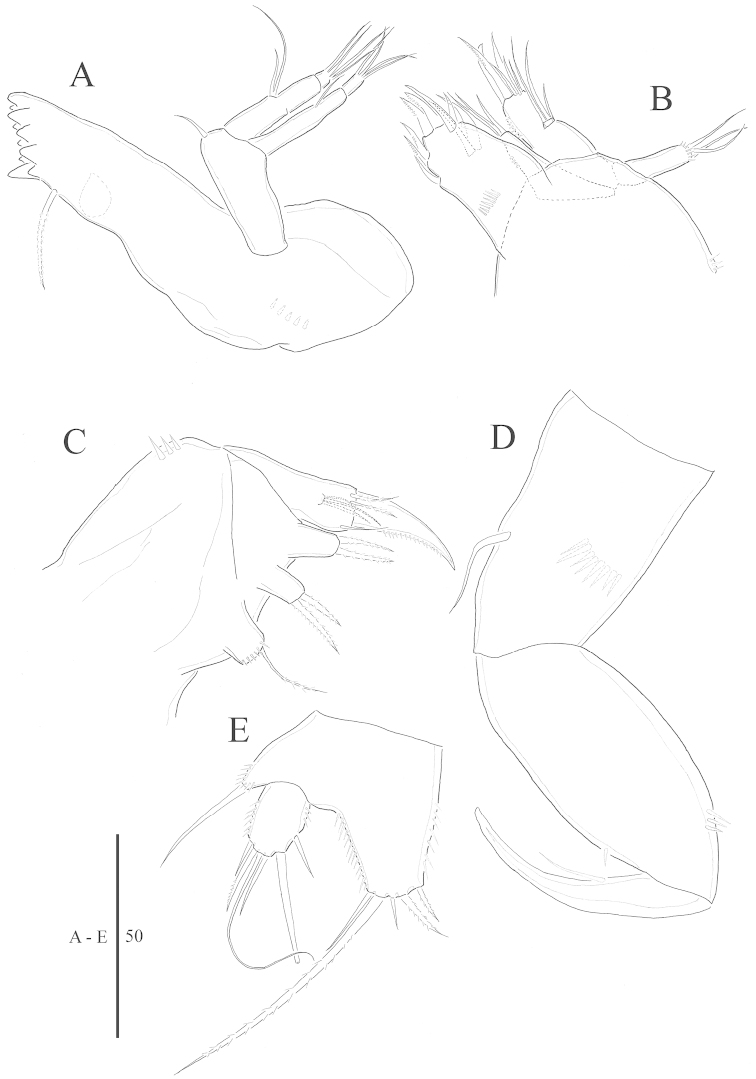
*Harpacticella jejuensis* sp. n. Female. **A** mandible **B** maxillule **C** maxilla **D** maxilliped **E** P5.

**Figure 4.**
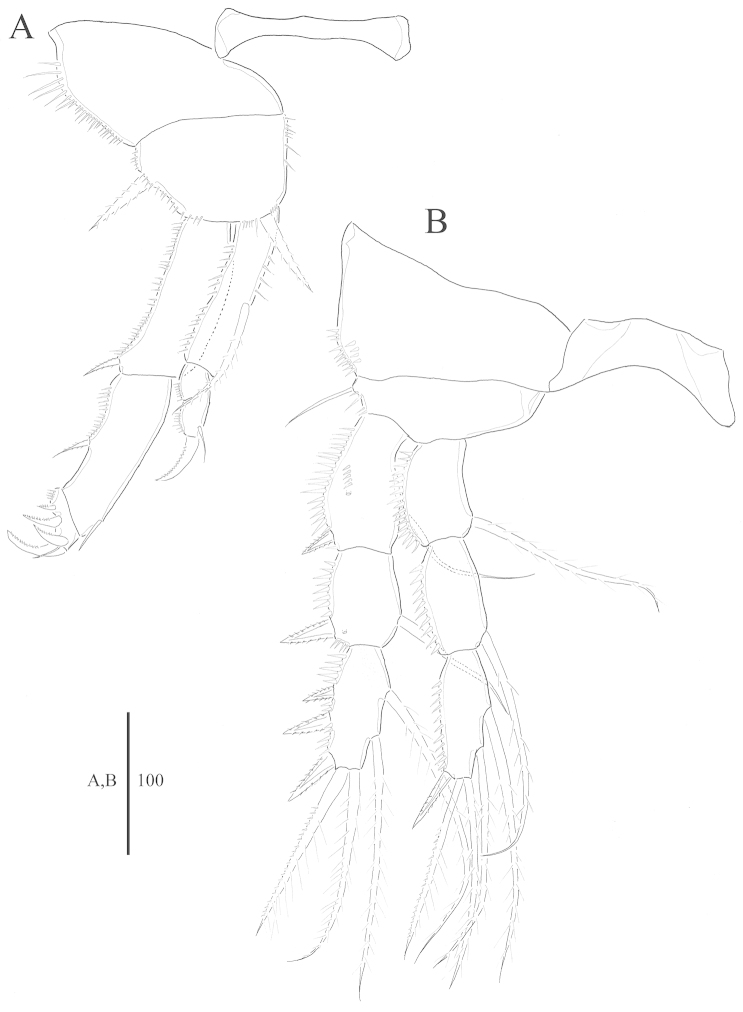
*Harpacticella jejuensis* sp. n. Female. **A** P1 **B** P2.

**Figure 5.**
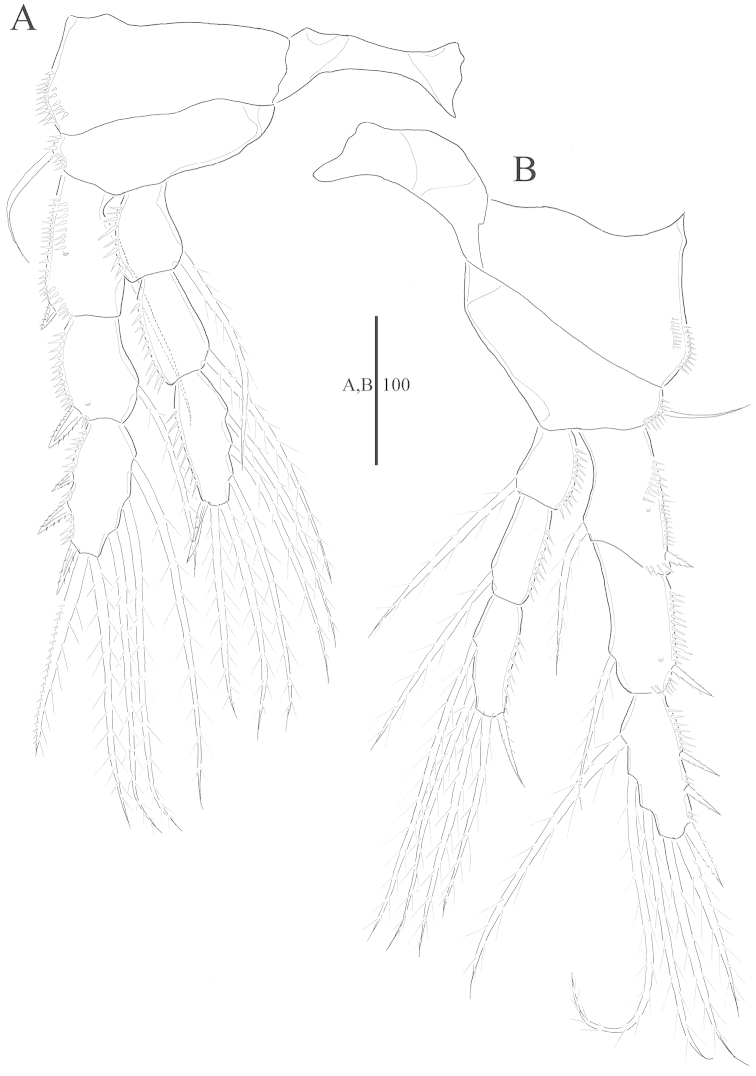
*Harpacticella jejuensis* sp. n. Female. **A** P3 **B** P4.

**Figure 6.**
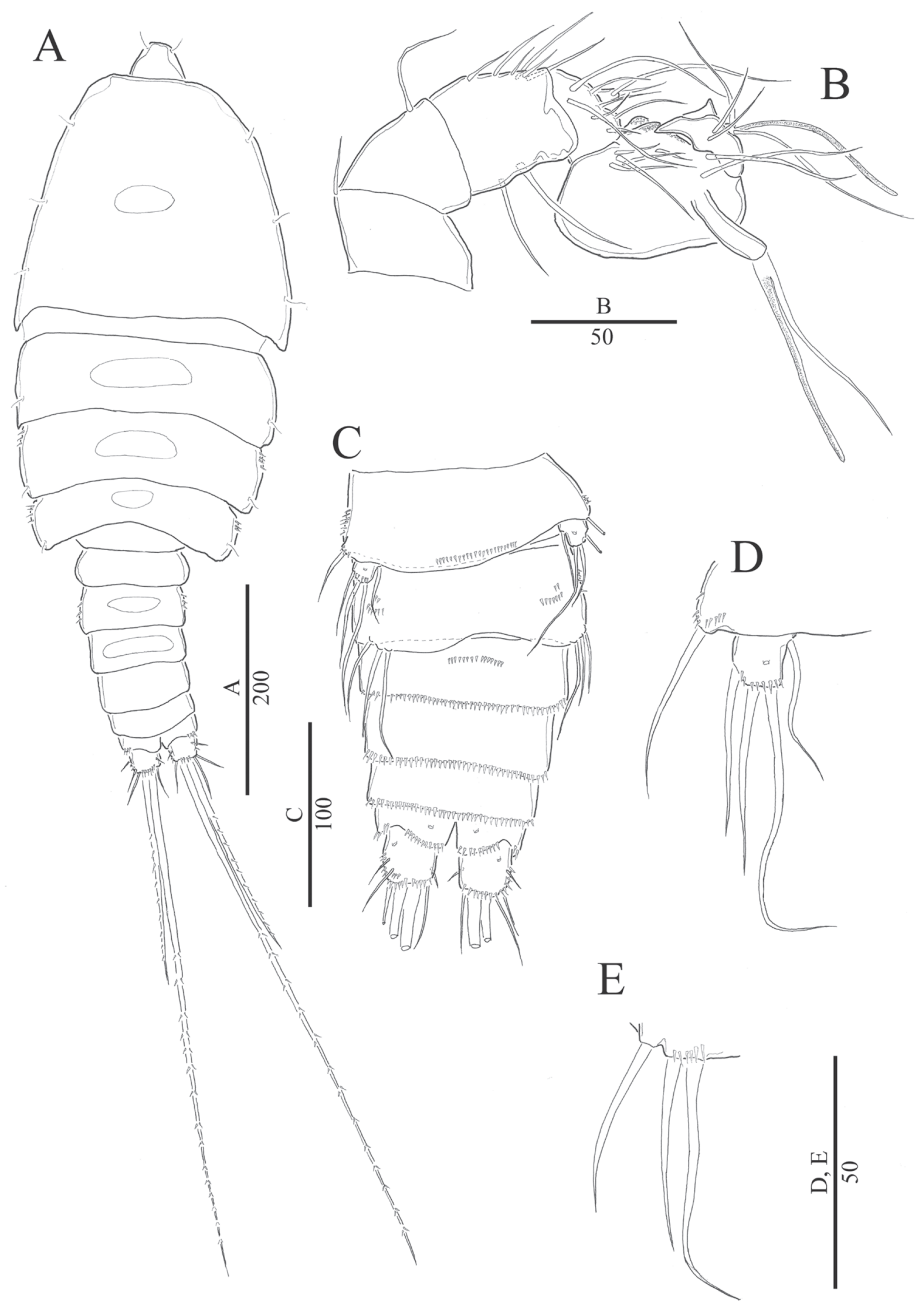
*Harpacticella jejuensis* sp. n. Male. **A** habitus, dorsal **B** antennule **C** urosome, ventral **D** P5 **E** P6.

Figs 1 , 2 , 3 , 4 , 5 , 6

##### Type locality.

A tidal pool (33°13.949'N; 126°30.653'E) on Beophwan beach, Seoguipo-shi, Jeju Island, Korea.

##### Materials examined.

Holotype: 1♀ (NIBRIV0000304111) in 70% ethanol from the type locality. Paratypes: 5♀♀ (NIBRIV0000304112) in 70% ethanol, 2♀♀ (NIBRIV0000304113 – NIBR0000304114) dissected on 11 and 10 slides, respectively, and 2♂♂ (NIBRIV0000304115 – NIBR0000304116) dissected on 11 and 2 slides, respectively. All from the type locality and collected by R. Jeong on 3 March 2013.

DNA-barcode (mtCOI) sequence and trace were submitted to GenBank (KM272559, 619 bp).

##### Description.

Female. Total body length 720–810 µm (mean = 759 µm; n = 10, measured from anterior margin of cephalosome to posterior margin of caudal rami). Maximum width at posterior margin of cephalosome (mean = 380 µm; n = 10). Urosome gradually tapering posteriorly. Body surface armed with some sensilla (Figs 1A–B).

Prosome (Fig. 1A–B) 4-segmented, comprising cephalosome and 3 free pedigerous somites. P1-bearing somite fused to cephalosome. Cephalosome (Fig. 1A) with few sensilla and smooth posterior margin. Other prosomites with few sensilla on dorsal and lateral surfaces. Dorsal tegumental windows of elongated oval shape, one on cephalosome and one on three succeeding prosomites; 2 windows on genital double somite (Fig. 1A). Pleural areas well developed and rounded, without lobate posterolateral angles.

Rostrum (Fig. 1A–C) well developed, trapezoid with smooth anterior apex, clearly defined at base. Dorsal surface smooth with pair of sensilla at apical margin.

Urosome (Figs 1A–B, 2C) 5-segmented, comprising the P5 somite, genital double somite, 2 free abdominal somites, and anal somite. All urosomites with row of spinules ventrally. P5-bearing somite with smooth dorsal surface and row of spinules along lateral margins.

**Figure 7.**
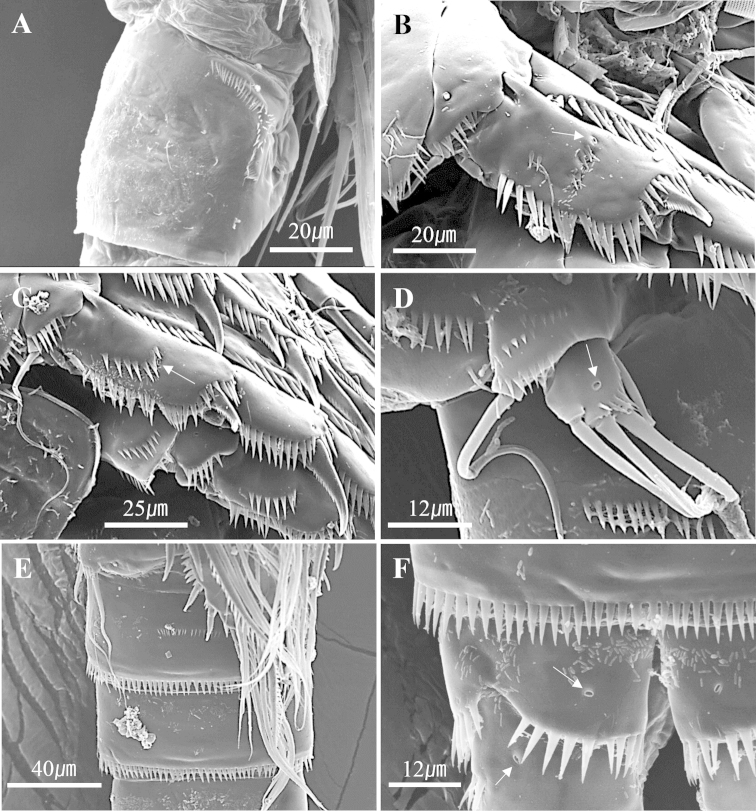
*Harpacticella jejuensis* sp. n. Scanning electron micrographs. Female: **A** genital double somite, lateral; Male. **B** P2 exp-1 **C** P3 exp-1 and 2 **D** P5 exp **E** 2 and 3 free abdominal somites, ventral **F** anal somite, ventral. Arrow indicates a pore on the surface.

Genital double somite (Figs 1A–B, 2C, 7A) subdivided by distinct chitinous structure laterally indicating original segmentation. Genital field located ventrally near anterior margin with copulatory pore positioned medially. P6 as small protuberance bearing 1 plumose seta.

Anal somite (Fig. 2C, D) without anal operculum, but with well-developed pseudoperculum arising from penultimate somite.

Caudal ramus (Figs 1A–B, 2D) wider than long; seta I inserted at half length of caudal ramus, ventrolaterally; lateral seta II longer than seta I, inserted close to distal outer corner; seta III as long as lateral seta II; apical seta IV unipinnate, slightly longer than urosome; apical seta V bipinnate, as long as whole body; apical seta VI similar in length to seta III; dorsal seta VII bare and bi-articulate at its base.

Antennule 7-segmented (Fig. 2A). Aesthetascs on segments 4 and 7. All setae slender and bare. Armature formula: 1-[1], 2-[9], 3-[7], 4-[2 + (1+1 ae)], 5-[2], 6-[1], 7-[3+1 acrothek]. Apical acrothek consisting of well-developed aesthetasc fused basally to two slender naked setae.

Antenna (Fig. 2B) 3-segmented, comprising coxa, allobasis, 1-segmented endopod, and 2-segmented exopod. Coxa small, bare. Allobasis elongated with two spinules on surface. First exp segment with bare seta on distal end; second segment 3 bare setae. Endopod with 2 spinules near proximal area, pinnate spine on dorsal surface, naked spine laterally; apically bare spine; 4 geniculate spiniform setae and bare spiniform seta, apically.

Mandible (Fig. 3A) with large coxa and well-developed gnathobase; cutting edge with 10 blunt teeth overlapping each other; accessory plumose seta at dorsal corner. Mandibular palp well developed. Basis with naked seta on lateral distal margin. Endopod 2-segmented; enp-1 with 2 juxtaposed setae at the middle of inner margin; enp-2 with 4 juxtaposed setae on distal end. Exopod 2-segmented; exp-1 with naked seta at the middle of inner margin; exp-2 with 4 juxtaposed setae on distal end.

Maxillule (Fig. 3B) with praecoxa arthrite with spinular row near proximal area; 2 spines on anterior and 1 on posterior surface subterminally; 2 unipinnate spines and 1 bare spine apically, 2 unipinnate spines and bare spine on lateral margin. Coxa with endite bearing 3 naked setae apically. Basis endite with row of spinules laterally; 3 spines and 3 bare setae apically. Endopod small and 1-segmented with 3 bare setae. Exopod elongated, with spinules and 3 setae apically.

Maxilla (Fig. 3C): syncoxa with 3 endites: proximal endite with 1 plumose seta apically; medial and distal endite each with 2 pinnate spines; basis with unipinnated claw: accessory armature consisting of 2 pinnated setae and bare seta. Endopod represented by small protuberance with 3 bare setae.

Maxilliped (Fig. 3D): syncoxa with bare seta and oblique row of spinules. Basis with spinule on inner margin and 3 spinules on outer margin. Endopod 1-segmented, forming strong spine with seta.

Swimming legs 1–4 (Figs 4A–B, 5A–B) biramous, P1–P4 with 3-segmented exopod and 3-segmented endopod; spinules along inner and outer margins as illustrated. Intercoxal sclerites well developed.

P1 (Fig. 4A): coxa with row of spinules along outer lateral margin. Basis shorter than wide, with strong outer seta; inner spine inserted near inner distal corner, with several spinules. Endopod shorter than exopod reaching about half of exp-2; end-1 with plumose seta at the middle of the inner margin; enp-2 small without ornamentation but a row of spinules along outer lateral margin; enp-3 longer than wide with pinnate claw distally and inner naked seta on distal inner margin. Exp-1 as long as exp-2, with spinules along outer margin and spine on distal outer corner; exp-2 with short naked seta on distal part of inner margin and pinnate spine at middle part of the outer margin; exp-3 short with 4 curved pinnate claws and bare seta.

P2 (Fig. 4B): coxa with 2 rows of spinules along outer lateral margin. Basis shorter than wide, with slender outer seta and spinules on outer lateral margin. Endopod as long as exopod; row of spinules along outer margin of each segment. Exopodal segments with row of spinules along outer margin; exp-1 with 2 rows of spinules and pore on anterior surface; exp-2 with pore on anterior surface.

P3 (Fig. 5A): coxa with 2 rows of spinules along outer lateral margin. Basis shorter than wide, with slender outer seta and spinules along outer lateral margin. Endopod reaching to middle of exp-3; row of spinules along outer margin of each segment. Exopodal segments with row of spinules along outer margin; exp-1 with 2 rows of spinules and pore on anterior surface; exp-2 with pore on anterior surface.

P4 (Fig. 5B): coxa with 2 rows of spinules along outer lateral margin. Basis shorter than wide, with slender outer seta and spinules on outer lateral margin. Endopod shorter than exopod, reaching to proximal half of exp-3; row of spinules along outer margin of each segment. Exopodal segments with row of spinules along outer margin; exp-1 with 2 rows of spinules and pore on anterior surface; exp-2 with pore on anterior surface.

Armature formulae as follows:

**Armature formulae:** 

Thoracopod	Exopod	Endopod
P1	0.1.050	1.0.110
P2	1.1.223	1.1.221
P3	1.1.323	1.1.321
P4	1.1.323	1.1.221

P5 (Fig. 3E): exopod and baseoendopod well separated. Baseoendopod with slender and bare outer lateral seta. Endopodal lobe larger than exopod and extended beyond distal margin of exopod; with 3 pinnate and 2 bare setae. Exopod oval, with 6 setae; rows of spinules along inner and outer margins.

Male. Total body length of examined samples 631–650 µm (mean = 643 µm; n = 5, measured from anterior margin of cephalosome to posterior margin of caudal rami). Greatest width at posterior margin of cephalosome. Cephalosome with sensilla along lateral margin. Other prosomites also with sensilla along lateral margin (Fig. 6A).

Prosome (Fig. 6A), 4-segmented, comprising cephalosome bearing first pedigerous somite and 3 free pedigerous somites. Cephalosome slightly narrower than in female, with smooth posterior margin. Prosomites 3 and 4 with some spinules along lateral proximal margin. Dorsal tegumental window elongated oval shape on cephalosome, three succeeding prosomites, and two genital somites (Fig. 6A). Rostrum well developed with pair of sensilla.

Urosome (Figs 6A, C, 7E) 6-segmented, with P5-bearing somite, genital somite, and 4 free abdominal somites. Free abdominal somites with rows of spinules ventrally. Caudal setae as in female. Sexual dimorphism in A1, P5, P6, and genital field.

Antennule (Fig. 6B) 6-segmented, chirocerate; geniculation between segment 5 and 6, segment 5 swollen and largest. Aesthetasc on segments 5 and 6. All setae slender and bare. Armature formula: 1-[1], 2-[1], 3-[8], 4-[9], 5-[7+(1+ae)], 6-[2+acrothek]. Apical acrothek with aesthetasc and 2 bare setae.

P5 (Figs 6C, D, 7D), baseoendopod fused medially, forming large transverse fig; each of them with slender and bare outer lateral seta. Exopod quadrangular, with 4 bare setae: inner, 2 terminal, outer bare setae.

P6 (Fig. 6C, E): small fig with 3 bare setae; inner seta longest, outer seta shortest; row of spinules near base of setae.

##### DNA barcode.

mitochondrial oxidase subunit I; partial cds; 619 bp

ACTTTATATCTTTTAAGGGGGATATGAGCGGGAGTTATGGGGGCGGCAATAAGAGTTATTATTCGGCTTGAATTAGGACAGCCTGGGACTTTAATTAAGGATGAGCAAATTTATAATGTTTTAGTGACTTCGCATGCTTTTATTATAATTTTCTTTATGGTTATACCAATTTTAATTGGGGGGTTTGGAAACTGGTTAGTTCCTTTAATATTAGGAGCTCCTGATATGGCCTTTCCTCGATTAAATAATTTGAGATTCTGATTTTTGATGCCCTCTCTTATATTAATAATTATTAGAAGAGTTGTTGAAGGCGGGGCAGGGACAGGGTGAACTGTTTACCCCCCTTTAAGAAGAAATTTAGCACATGCAGGAGGCTCGGTGGATTTAGTAATTTTTTCTTTACATTTAGCAGGAGTTTCTTCCTTATTAGGGGCTGTAAATTTTATTAGGACTTTAAGAAATCTTCGAGTATTCGGGATGTATTTTGACCAAGTGCCGTTATTTTGTTGATCTGTCTTGGTTACAGCTGTTCTATTACTTTTATCACTGCCTGTATTAGCGGGGGCAATTACTATATTGTTGACCGATCGAAACATTAATTCAAGCTTCTATGATGTTA

##### Etymology.

The specific name refers to the type locality of Jeju Island, Korea.

## Discussion

The new species clearly fits in the genus *Harpacticella* based on the combination of following character sets: a) 7-segmented antennule in the female, b) 2-segmented antennary exopod, c) 3-segmented P1 endopod and exopod, d) only one seta on the inner edge of P2 enp-2 and e) spiniform outer spine of exp-3 P3 and P4 (Table 1). *Harpacticella jejuensis* sp. n. is closely related to *Harpacticella itoi* Chang & Kim, 1991 based on the length of P1 exp-1 and enp-1, the length of P1 exp-1 and exp-2, and the four setae on the P5 exopod of males. However, *Harpacticella jejuensis* can be distinguished from *Harpacticella itoi* by the following distinctive characters: (1) six setae on the P5 exopod of females (Fig. 2E) compared to five setae in *Harpacticella itoi* (see Fig. 3A; Chang and Kim 1991; this character is a unique character within the genus); (2) one bare seta on inner margin of P1 exp-2 (Fig. 4A), which is absent in *Harpacticella itoi* (see Fig. 1C; Chang and Kim 1991); (3) P1 endopod much shorter than exopod (ratio = 0.64:1), but in *Harpacticella itoi* it is as long as exopod (see Fig. 1C; Chang and Kim 1991); (4) naked seta on proximal inner margin of male P5 (Fig. 6D), but plumose-type in *Harpacticella itoi* (see Fig. 3E; Chang and Kim 1991); (5) naked seta on proximal inner margin of male’s P5 is three times longer than length of male’s P5 exopod (Fig. 6D), but it is almost two times longer in *Harpacticella itoi* (see Fig. 3E; Chang and Kim 1991).

**Table 1.** Morphological comparison of species in the genus *Harpacticella* Sars, 1908.

Characters	*Harpacticella amurensis*	*Harpacticella inopinata*	*Harpacticella itoi*	*Harpacticella lacustris*	*Harpacticella oceanica*	*Harpacticella paradoxa*	*Harpacticella jejuensis*
**A2**							
No. of exp segments	1	2	2	2	2	2	2
No. of exopodal setae	3	3	4	3	4	4	4
**Md**							
No. of basal setae	unknown	2	1	2	2	1	1
**P1**							
Length of exp / enp	1.3	1.4	1.3	1.3	1.2	1.5	1.5
Length of exp-1 / enp-1	1.1	1.4	0.9	0.9	0.9	1.1	1.1
**P2**							
Length of exp / enp	1.2	1	1	1.1	1.2	1	1
Exp	1.1.323	1.1.323	1.1.323	1.1.323	1.1.323	1.1.323	1.1.323
Enp	1.1.221	1.1.221	1.1.221	1.1.221	1.1.221	1.1.221	1.1.221
**P3**							
Exp	1.1.323	1.1.323	1.1.323	1.1.323	1.1.323	1.1.323	1.1.323
Enp	1.1.321	1.1.321	1.1.321	1.1.321	1.1.321	1.1.321	1.1.321
**P4**							
Exp	1.1.323	1.1.323	1.1.323	1.1.323	1.1.323	1.1.323	1.1.323
Enp	1.1.221	1.1.221	1.1.221	1.1.221	1.1.221	1.1.221	1.1.221
**P5 female**							
No. of exopodal seta	4	5	7	5	5	5	6
Length of exp / enp	0.6	0.6	0.7	0.7	0.4	0.4	0.7
**P5 male**							
No. of exopodal seta	unknown	3	4	3	4	3	4
**Body size** (µm)							
Female	700	800	650	650	620	850	780
Male	unknown	700	530	550	560	unknown	650
**Type locality**	Amur River (Borutzky 1952)	Lake Baikal (Sars 1908)	Tamjin River, Korea (Chang and Kim 1991)	Chilka Lake near Calcutta, India (Sewell 1924)	Bonin Island, Japan (Itô 1977)	Talifu Lake in Yunnan Province, China (Brehm 1924)	Seogwipo in Jeju Island, Korea

**Figure 8.**
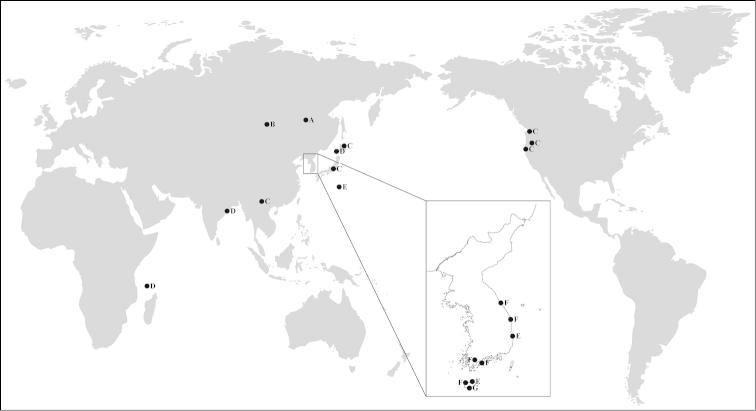
Distribution of *Harpacticella* species. **A** *Harpacticella amurensis* **B** *Harpacticella inopinata* **C** *Harpacticella paradoxa* **D** *Harpacticella lacustris* **E** *Harpacticella oceanica* **F** *Harpacticella itoi* **G** *Harpacticella jejuensis* sp. n.

*Harpacticella* species have a wide distribution ranging from freshwater to true marine environments, and have been found in Asian waters, American waters, and the Aldabra Atoll in the Indian Ocean (Fig. 8). All species in this genus except *Harpacticella amurensis* Borutzky, 1952 and *Harpacticella inopinata* Sars, 1908 have been recorded from at least two localities; the former two species have been recorded only in the type locality (Itô and Kikuchi 1977; Evstigneeva 1993). *Harpacticella itoi* Chang & Kim, 1991 has been found in several locations in the southeastern part of Korean peninsula and *Harpacticella oceanica* Itô, 1977 was documented in Korean and Japanese marine waters (Itô 1977; Chang and Kim 1991; Song and Chang 1993). *Harpacticella lacustris* Sewell, 1924 has a discontinuous distribution and has been recorded in India, China, and Japan (Sewell 1924; Wells and McKenzie 1973; Ishida 1989b). *Harpacticella paradoxa* (Brehm, 1924) is the most ubiquitous species; it has been recorded in China, Japan, and the northwest coast of the USA (Brehm 1924; Pesta 1930; Itô and Kikuchi 1977; Ishida 1987; Ishida 1989a; Ishida 2003; Ishida et al. 2004; Cordell et al. 2007). Cordell et al. (2007) suggested that the introduction of *Harpacticella paradoxa* may have been due to anthropogenic factors such as ballast waters. Small marine invertebrates have been shown to be introduced into new marine ecosystems via ballast water (Orsi and Ohtsuka 1999). Recently, molecular approaches have been used to determine the origin of these invasive organisms (Le Roux and Wieczorek 2009; Simon et al. 2011)

DNA barcoding is an efficient tool to identify species, especially morphologically similar species (Floyd et al. 2002; Hebert et al. 2003; Guidetti et al. 2005; Bhadury et al. 2006). This barcode can also be used for biogeographical analysis of invasive or widely distributed species (Garrick et al. 2004). We obtained a 619-bp partial sequence of mtCOI (KM272559) for use in future studies; no sequences have been obtained from congeners to date, even though it would be interesting to determine the phylogenetic relationships among congeners based on analysis of mtCOI sequences.

### Key to Species in the Genus *Harpacticella*, 1908

**Table:** 

1	A2 exp with 3 setae	**2**
–	A2 exp with 4 setae	**4**
2	A2 exp 1-segmented	***Harpacticella amurensis***
–	A2 exp 2-segmented	**3**
3	P1 exp-1 much longer than P1 enp-1	***Harpacticella inopinata***
–	P1 exp-1 as long as P1 enp-1	***Harpacticella lacustris***
4	Md basis with 1 seta; P2 exp as long as P2 enp	**5**
–	Md basis with 2 setae; P2 exp longer than P2 enp	***Harpacticella oceanica***
5	P5 exp of female with 5 setae; P5 exp of male with 3 setae	***Harpacticella paradoxa***
–	P5 exp of female with 6 setae; P5 exp of male with 4 setae	***Harpacticella jejuensis* sp. n.**
–	P5 exp of female with 7 setae; P5 exp of male with 4 setae	***Harpacticella itoi***

## Supplementary Material

XML Treatment for Harpacticella jejuensis
